# *Exophiala* species in household environments and their antifungal resistance profile

**DOI:** 10.1038/s41598-024-68166-4

**Published:** 2024-07-31

**Authors:** Nahid Kondori, Daniel Jaén-Luchoro, Roger Karlsson, Bahman Abedzaedeh, Helena Hammarström, Bodil Jönsson

**Affiliations:** 1 Department of Infectious Diseases, Institute of Biomedicine, Sahlgrenska Academy, University of Gothenburg, Box 7193, 402 34 Gothenburg, Sweden; 2 Department of Clinical Microbiology, Sahlgrenska University Hospital, Region Västra Götaland, Gothenburg, Sweden; 3 Nanoxis Consulting AB, Gothenburg, Sweden; 4 Department of Infectious Diseases, Sahlgrenska University Hospital, Region Västra Götaland, Gothenburg, Sweden

**Keywords:** *Exophiala dermatitidis*, Filamentous fungi, Indoor environments, Black fungi, Antifungal agents, Fungi

## Abstract

The black fungus *Exophiala* causes a wide range of infections from superficial to subcutaneous, but also invasive fungal infections in immunocompromised patients as well as healthy individuals. In addition, *Exophiala*, is a common colonizer of the air ways of patients with cystic fibrosis. However, the source of infection and mode of transmission is still unclear. The aim of this study was to investigate the presence of *Exophiala* in samples collected from Swedish indoor environments. We found that the *Exophiala* species were commonly found in dishwashers and that *Exophiala dermatitidis* was the most common *Exophiala* species, being identified in 70% (26 out of the 37) of samples. Almost all *E. dermatitidis* isolates had the ability to grow at 42 °C (*P* = 0.0002) and were catalase positive. Voriconazole and posaconazole exhibited the lowest MICs, while caspofungin and anidulafungin lack the antifungal activities in vitro. Future studies are needed to illuminate the transmission mode of the fungi.

## Introduction

Fungal diseases kill more than 1.5 million people and affect over one billion people ^1^. Although *Candida*, *Aspergillus*, *Pneumocystis* and *Cryptococcus* spp. are the most common causes of severe fungal infections in Europe, there has been an increase in the incidence of infections caused by rare filamentous fungi ^2^. The black fungus *Exophiala* causes a wide range of infections, including superficial, subcutaneous, and invasive fungal infections in immunocompromised patients as well as healthy individuals ^3^. In addition, *Exophiala*, is a common colonizer of the airways of patients with cystic fibrosis, and the fungus has been isolated from sputum of 18% of Swedish patients ^4,5^. The clinical importance of *E. dermatitidis* in cystic fibrosis patients is still unclear. However, the fungus was found to trigger the immune system to produce specific IgG antibodies against *Exophiala* ^6^. Furthermore, it was observed that the presence of *Exophiala* in the airways was negatively associated with lung function ^6^. *E. dermatitidis* has demonstrated remarkable neurotropism, acting as a true pathogen, infecting mainly healthy individuals ^7^. Several cases of meningitis have been reported in the United States after steroid injections contaminated with *E. dermatitidis* ^8^. However, the source of infection and mode of transmission is still unclear. *Exophiala* has been isolated from natural habitats such as tropical rainforests and glaciers. This fungus has also frequently been isolated from human-made high humidity environments rich in aromatic hydrocarbons such, as steam baths and dishwashers ^7,9^. The presence of *Exophiala* in Nordic indoor environments has not previously been studied.

The aims of this study were to investigate the presence of *Exophiala* spp in indoor environments and to determine their physiological activity and antifungal susceptibility in a Nordic setting. The presence of *Exophiala* species in human-derived environments may provide valuable information regarding possible sources of infection and mode of transmission to patients.

## Results

### Isolation of *Exophiala* from household environment

A total of 388 samples were analyzed, of which 37 (10%) were culture positive for *Exophiala* species. Molecular identification of isolates revealed that *Exophiala dermatitidis* was the most common *Exophiala* species, being identified in 26 out of the 37 samples. The highest number of *Exophiala* species were recovered from dishwashers followed by bathrooms (Table 1). *E. dermatitidis* was predominantly found in samples from dishwashers, representing 12 out of 13 isolates from these samples*.*

**Table 1:** Number of isolated *Exophiala* spp from various areas in a household.

Local	Number of Samples n (% of total)	*Exophiala dermatitidis* n (% of total)	non -dermatitidis *Exophiala* n (% of total)	Total number of samples positive for *Exophiala* spp
Dishwasher	70 (18)	12 (17)	1 (1.4)	13 (18.6)
Bathroom	84 (21.6)	6 (7)	4 (4.8)	10 (11.9)
Coffee machine	76 (19.6)	4 (5.2)	3 (3.9)	7 (9.2)
Faucet opening	83 (21.4)	3 (3.6)	3 (3.6)	6 (7.2)
Washing machine	75 (19.3)	1 (1.3)	0	1 (1.3)
Total	388	26 (6.7)	11 (2.8)	37 (9.5)

Other *Exophiala* species were also isolated from the samples (Table 2). Briefly, *E. pisciphila* (n = 3), *E. phaeomuriformis* (n = 2), *E. alcalophila* (n = 1), *E. mesophila* (n = 1), *E. spinifera* (n = 1), *E. xenobiotica* (n = 1) and one unidentified *Exophiala* species were found. Non-dermatitidis *Exophiala* were often isolated from samples collected from bathrooms (n = 4) followed by coffee machines (n = 3) and faucet opening (n = 3).

**Table 2:** Non-dermatitidis *Exophiala* species isolated from areas in a household.

	Dishwasher	Bathroom	Coffee machine	Faucet opening	Washing machine
*Exophiala alcalophila*				1	
*Exophiala mesophila*				1	
*Exophiala phaeomuriformis*	1		1		
*Exophiala pisciphila*		2		1	
*Exophiala spinifera*		1			
*Exophiala xenobiotica*			1		
*Exophiala species*		1	1		
Total	1	4	3	3	0

### The physiological activity of *Exophiala* species

Almost all of the *E. dermatitidis* isolates, but only two of the non-dermatitidis *Exophiala* isolates (*E. spinifera* and *E. mesophila*), had the ability to grow at 42 °C (*P* = 0.0002) (Table 3). All isolates, from both *E. dermatitidis* and non-dermatitidis, were catalase positive, and the majority of isolates were urease positive. Esculin hydrolase activity was found only in 8 non-dermatitidis *Exophiala* isolates (80). None of the *Exophiala* isolates showed oxidase activity.

**Table 3:** The physiological activitities of *Exophiala dermatitidis* and non-Exophiala *dermatitidis* species.

	*Exophiala dermatitidis* n = 25 (%)	Non-dermatitidis *Exophiala* n = 10 (%)	P
DNase	0	0	
Esculin	0	8 (80%)†	0.0002
Catalase	25 (100)	10 (100)	
Oxidase	0	0	
Urease	14 (56)	10 (100)	0.08
42 °C	23 (92)	2 (22) ‡	0.0002

^†^ Two isolates of *Exophiala phaeomuriformis* were esculin negative. ‡ *Exophiala spinifera* and *Exophiala mesophila* were positive for grow at 42 °C.

### The susceptibility of *Exophiala* to anti-fungal drugs

**Figure 1:**
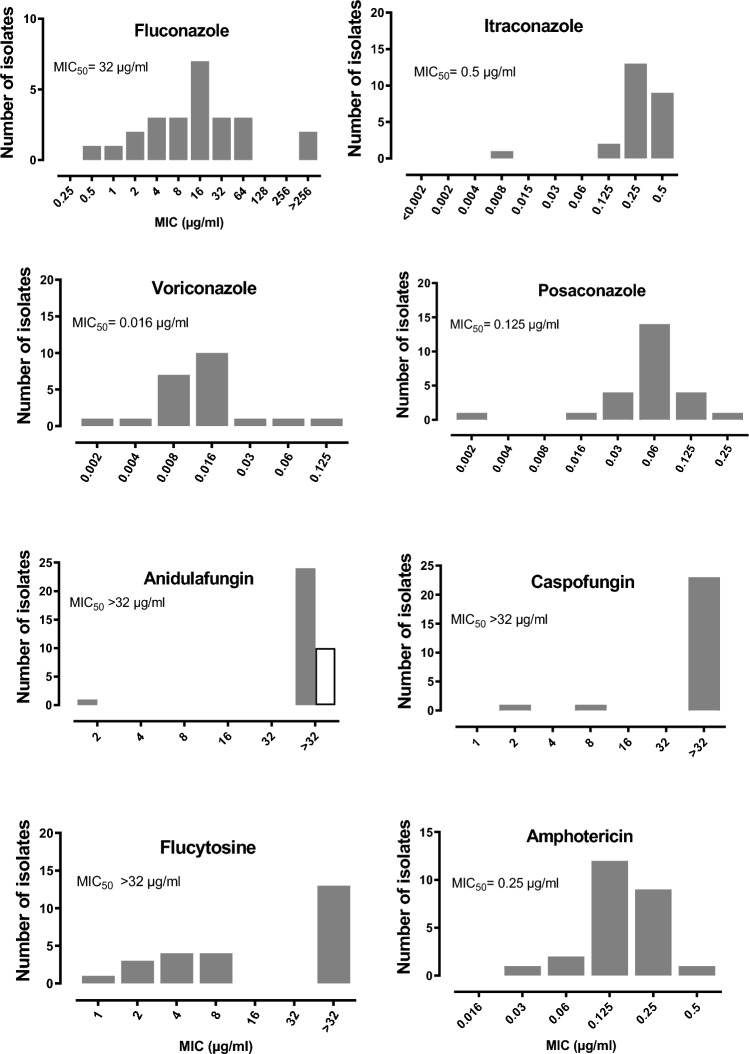
Minimum inhibitory concentration (MIC) of eight antifungal agents to environmental *Exophiala dermatitidis*. The MIC_50_ represents the antifungal concentration that inhibited the growth of 50% of the *Exophiala* isolates.

The minmum inhibitory concentration (MIC) ranges, including the MIC_50_ values of antifungal agents to *E. dermatitidis*, are shown in Fig. 1. Voriconazole and posaconazole exhibited the lowest MICs, followed by itraconazole and amphotericin. Caspofungin and anidulafungin had very broad range of MICs to *E. dermatitidis* (Fig. 1). Caspofungin and anidulafungin did not show any antifungal activity regarding the non-dermatitidis *Exophiala* isolates (Table 4). A higher MIC for fluconazole tested in this study were found for non-dermatitidis *Exophiala* (> 256 µg/ml) compared with *E. dermatitidis* (32 µg/ml).

**Table 4:** Minimum inhibitory concentration (MIC) of eight antifungal agents to environmental non-dermatitidis *Exophiala* species.

Antifungal agents	*Exophiala alcalophila* (n = 1)	*Exophiala mesophila* (n = 2)	*Exophiala phaeomuriformis* (n = 2)	*Exophiala pisciphila* (n = 3)	*Exophiala spinifera* (n = 1)	*Exophiala xenobiotica* (n = 1)	*Exophiala species* (n = 1)
	MIC µg/ml						
Fluconazole	> 256	> 256	32- > 256	> 256	32	> 256	> 256
Itraconazole	0.016	0.25	0.25 -0.5	0.064–0.25	0.064	0.016	0.016
Voriconazole	0.125	0.125	0.008- 0.125	0.002- 0.125	0.125	0.008	0.008
Posaconazole	0.032	0.016	0.008- 0.25	0.002–0.032	0.25	0.004	0.004
Caspofungin	> 32	> 32	> 32	> 32	> 32	> 32	> 32
Anidulafungin	> 32	> 32	> 32	> 32	> 32	> 32	> 32
Amphotericin	0.064	0.125	0.125- 0.5	0.016–0.125	0.125	0.5	0.5
5-Flucytosine	> 32	> 32	1- 4	> 32	> 32	> 32	> 32

## Discussion

We found that almost 10% of the samples collected from indoor environments were positive for black fungus, being *E. dermatitidis* the dominant species. The black fungi *E. dermatitidis* has been found to be a frequent colonizer of patients with cystic fibrosis ^4^, causing airway infection/colonization in this patient group ^6^. Little is known about the natural habitat of *Exophiala* or possible infection routes. Various studies reported the presence of *E. dermatitidis* in tropical rainforests, steam baths, bathrooms, kitchen sinks and dishwashers ^9–12^. However, the presence of *Exophiala* in the human population of the Nordic countries has not been previously investigated. In the present study we analyzed 388 indoors samples of which 10% were positive for black fungus. The fungus was more frequently isolated from dishwashers, being present in 17% of the dishwashers analyzed. This finding agrees with a previous study by Dogen et al. ^10^ who showed that *E. dermatitidis* was the most common fungi isolated from dishwashers in Turkish household, being detected in 24 out of 158 (15%) dishwashers analyzed in their study. Earlier studies showed that the highest frequencies of *E. dermatitidis* in indoor environments are found in steam baths and dishwashers, where high temperatures, water and changing pH combine ^7^. In our hands, only one sample collected from a washing machine was positive, which correlates with a previous study in which the number of *E. dermatitidis* was very low or absent in washing machines ^10,13^.

All *E. dermatitidis* isolated in this study had catalase activity. Catalases are antioxidant enzymes that are found in aerobic microorganisms and protect the cells from oxidative damages. Catalase activities have previously been reported for *Candida albicans, Cryptococcus neoformans* and *Aspergillus species*. Catalase production may be a virulence factor of *Candida albicans* and *Cryptococcus neoformans* ^14^. Sav et al. reported catalase activity in all environmental and clinical *E. dermatitidis* ^15^.

*E. dermatitidis* is a pathogenic fungus, which can cause neutropenic infections in humans. *E. dermatitidis* poses enormous ecological plasticity, which might play a role in fungus distribution and enables the transition of the fungus from the environment to a human host ^16^. This thermotolerant fungus has been found in high temperature man-made habitats such as sauna facilities and dishwashers ^9,11^. The majority of *E. dermatitidis* isolates in this study were thermotolerant and are able to grow at 42 °C, while only two non-dermatitidis *Exophiala* species were able to grow at this temperature.

The antifungal susceptibility testing exhibited that voriconazole and posaconazole followed by amphotericin and itraconazole, were the most effective antifungals against *E. dermatitidis*, which are in concordance with previous published studies ^4,17^. Echinocandines (anidulafungin and caspofungin) and 5-flucytosine had no effect on *Exophiala* species, in vitro.

In conclusion, *Exophiala* species are found in humid common indoor environments and *E. dermatitidis* is the most common species. This thermotolerant fungus was frequently found in dishwashers. Its ability to produces various enzymes and grow at higher temperature may play an important role in its pathogenicity. The fungus also exhibits reduced susceptibility to several antifungal agents. Future studies are needed to clarify the infection’s sources and mode of transmission.

## Material and methods

### Sampling

From January to September 2016, a total of 388 samples from 89 households located in Region Västra Götaland, Gothenburg, were collected. The samples were collected from faucet opening (n = 83), coffee machines (n = 76), dishwashers (n = 70), washing machines (n = 75), and bathrooms (floor and walls, n = 84) using sterile cotton swabs. The samples were cultivated on Sabouraud dextrose agar plates and erythritol-chloramphenicol (ECA) agar plates. All plates were incubated for up to 3 weeks at 30 °C. The plates were checked for growth twice a week.

### Isolation and identification of *Exophiala* species

Brownish colonies were cultivated on Sabouraud agar plates for confirmation of purity and maintenance of the isolates. Pigmented fungi were identified to the species level using morphological criteria, and molecular identification as previously described ^4^.

### Physiological activity

*DNase.* The extracellular DNase activity was analyzed by using a DNase test agar plate as previously described. The microorganism suspension was cultured on DNase medium and incubated at 30 °C for 7 days. The plates were then flooded with 1N hydrochloric acid. After 5 min, the excess fluid was removed and clear zones around the colonies were measured ^18^. *Staphylococcus aureus* CCUG 17,621 was used as control.

*Esculin activity.* The ability of fungi to hydrolyse esculin (a coumarin glucoside) into glucose is measured by incubation of a fungal colony a the medium (3 ml) composed of esculin 1 mg/ml and ferric citrate 0.5 mg/ml in peptone water. The fungal suspension was incubated at 37 °C for 48 h. The production of a black coloured component in medium is considered as a positive reaction ^19^.

*Catalase activity.* A colony of fungal isolate was transferred on to a glass microscope slide and mixed with 20 µl of hydrogen peroxidase at room temperature. The formation of air bubbles was considered as a positive catalase activity. *Staphylococcus aureus* CCUG 17,621 was used as a control.

*Oxidase.* Peroxidase (20 µl) was added to a glass microscope slide. A small portion of a fungal colony was removed from the agar surface and mixed with oxidase substrate. The formation of a color change to deep blue or purple within 10 s were considered as a positive reaction. *Staphylococcus aureus* CCUG 17621 was used as a control.

*Urease.* The fungal isolates were cultured on Urease agar medium and incubated for three days at 25 °C. The fungal isolates were considered urease positive when a pinkish discoloration in medium was observed.

*Thermotolerance.* The fungal tolerance to higher temperatures was studied by incubation of the fungal isolates on Sabouarud agar plates at 42 °C for three days.

### Antifungal susceptibility testing

Antifungal susceptibilities were determined by Etest (bioMerieux, Marcy-l'Étoile, France) as previously described^4^. The concentrations of the antifungal agents on Etest strips ranged from 0.002 to 256 μg/mL for fluconazole and from 0.002 to 32 μg/mL for itraconazole, voriconazole, posaconazole, caspofungin, amphotericin B, and flucytosine. Briefly, fungal colonies were suspended in 0.85% saline solution, and the turbidity was adjusted to 0.5 on the McFarland scale (1 × 10^6^ to 5 × 10^6^ CFU/mL). The suspensions were further diluted 1:5 in 0.85% saline and flooded on agar plates consisting of RPMI 1640 medium supplemented with 2% glucose and buffered with morpholinopropanesulfonic acid. Excess fluid was removed, and the plates were allowed to dry at room temperature before the Etest strips were applied and incubated for 72 h at 37 °C. The MICs of antifungal agents were read as the lowest concentration at which the border of the elliptical inhibition zone intercepted the scale of the strips. The MIC_50_ results are the concentrations of each antifungal agent necessary to inhibit the growth of 50% of the fungal isolates.

### Statistical analysis

The microbiological data was analyzed by Fisher’s exact test using GraphPad Prism, version 9 (GraphPad, San Diego, CA, USA).

### Ethics approval

Ethical approval was not required for this study based on the nature of the manuscript.

## Data Availability

The datasets present in the current study could be available through the corresponding author.
